# Promoting Wound Regeneration Through Targeted Suppression of Chronic Inflammation with Active Molecular Chitosan

**DOI:** 10.3390/gels12050384

**Published:** 2026-05-01

**Authors:** Ji Eun Yoo, Zio Song, Yong Hyun Lee, Jae Kweon Park

**Affiliations:** Department of Life Sciences, Gachon University, Seongnamdaero 1342, Seongnam-si 461-701, Gyeonggi-do, Republic of Korea

**Keywords:** antimicrobial, anti-inflammatory, chitosan, hydrogels, wound healing

## Abstract

This study aimed to investigate the wound-healing mechanisms of chitosan with a defined molecular weight (MW) and degree of deacetylation (DD), and to explore its potential in hydrogel formulations, optimized for enhanced antibacterial performance. An active molecular chitosan (AMC) was prepared via enzymatic treatment to target a specific MW range with excellent biological activity. The antibacterial, anti-inflammatory, and wound-healing effects of AMC-based hydrogels were evaluated. Given AMC’s antibacterial activity against vancomycin-resistant *Staphylococcus aureus* (VRSA), its anti-inflammatory effects were also evaluated in full-thickness wounds in BALB/c nude mice. Anti-inflammatory effects were assessed using ELISA and immunohistochemical staining to measure levels of IL-1β, IL-4, IL-6, IL-10, and TNF-α. AMC treatment significantly reduced wound size and suppressed inflammatory cytokine production. These results suggest that hydrogels containing AMC may enhance both antibacterial and anti-inflammatory properties, potentially promoting wound healing.

## 1. Introduction

Wounds from daily accidents can progress to chronic states when the physiological balance of the healing process is disrupted, prolonging inflammatory phases. Chronic wounds are frequently colonized or infected by pathogenic microorganisms, which not only impede the regenerative cascade but also exacerbate underlying systemic conditions [1,2]. Pathological hallmarks of these wounds include impaired proliferation of dermal fibroblasts, reduced synthesis of extracellular matrix components and proteins, and diminished bioavailability of critical growth factors [3,4]. To address these challenges, significant research has focused on developing biocompatible, non-toxic natural materials with potent antimicrobial and regenerative properties, particularly as sophisticated drug delivery vehicles [5,6]. Among various natural polymers, chitosan has garnered attention for its molecular weight-dependent biological activities, including antioxidant, anti-inflammatory, and antimicrobial effects, positioning it as a versatile matrix for hydrogels and targeted delivery systems.

Chitosan, a linear polysaccharide composed of D-glucosamine (GlcN) units, has been extensively utilized in biomedical applications due to its multifaceted biological activities [7,8]. Derived from the deacetylation of chitin—the second most abundant natural polymer after cellulose—it shares a structural framework of ß-(1,4)-linked *N*-acetylglucosamine (GlcNAc). The biological efficacy of both chitin and chitosan is critically governed by their molecular weight (MW) and degree of deacetylation (DD) [9,10]. Unlike the predominantly insoluble chitin macromolecule, whose biological activity is often restricted to low-molecular-weight derivatives (<1 kDa), chitosan exhibits superior solubility in organic acids such as acetic and lactic acid. Consequently, academic interest has shifted toward characterizing the physicochemical properties of chitosan. Systematic efforts to identify the optimal MW and DD for pharmacological and pharmaceutical purposes have further elucidated its molecular-driven functionalities, including immuno-enhancing [11], antimicrobial [12], antioxidant [13], and antineoplastic activities [14].

Despite extensive research on the biological activities of chitosan, inconsistent results persist due to inherent variations in its MW and DD. For instance, high-molecular-weight chitosan (HMWC) exceeding 1000 kDa has demonstrated significant anti-inflammatory and immunomodulatory potential [15]. However, the clinical translation of HMWC is frequently hampered by its poor digestibility and limited bioavailability within the human gastrointestinal tract [16,17]. Furthermore, the biochemical requirements for HMWC in in vitro settings—such as the necessity for specific organic solvents and the maintenance of a low pH to ensure solubility—restrict its versatility in diverse experimental models. Consequently, the standardization of chitosan to well-defined, low-molecular-weight or specific MW fractions remains a critical prerequisite for the rigorous assessment of its pharmacological efficacy and for ensuring the reproducibility of bioactivity data.

Chitooligosaccharides (COS), typically comprising (GlcN)_2_ to (GlcN)_6_, face significant scalability challenges for industrial-scale production. To address this, our group has developed an optimized enzymatic process to mass-produce hydrolysates with a MW range of 1–3 kDa, which exhibit potent antimicrobial, antioxidant, and anti-inflammatory activities [13,18]. To distinguish this specific fraction from conventional COS (<1 kDa), we designated it as “Active Molecular Chitosan (AMC).” While efficient MW control for AMC production has been established, the purification of individual oligomers for clinical standardization remains an ongoing challenge. In this study, we formulated AMC-incorporated hydrogels and evaluated their therapeutic efficacy in wound healing, focusing on their capacity to prevent secondary infections and modulate inflammatory responses. Current polymer-based hydrogels, such as those derived from cellulose and hyaluronic acid, are favored in clinical settings for their high biocompatibility and low toxicity. However, these materials primarily serve as passive scaffolds and lack inherent antimicrobial properties. This deficiency often leads to critical clinical failures, including an inability to prevent secondary infections and a lack of active signals required for rapid tissue regeneration. Consequently, their effectiveness in managing chronic or infected wounds remains insufficient, necessitating the development of next-generation biologically active dressings. Among natural biopolymers, chitosan stands out for its unique ability to disrupt microbial cell walls through cationic interaction while simultaneously promoting collagen synthesis and cell proliferation. Despite these advantages, its pharmaceutical translation is severely hindered by the challenge of selective MW control. The biological performance of chitosan is inextricably linked to its MW distribution [19,20]; however, traditional production methods often yield wide polydispersity and inconsistent purity, leading to unpredictable clinical outcomes.

The production of water-soluble, low-molecular-weight COS has historically relied on diverse physical, chemical, and enzymatic methods. However, as demand for biocompatibility and environmental sustainability grows, enzymatic hydrolysis has emerged as the preferred approach, using both specific chitosanases and non-specific enzymes such as cellulases [20,21]. Despite this shift, the field lacks a standardized processing framework because of the inherent insolubility of HMWC, which complicates the precise determination of enzymatic kinetics, including Vmax and Km. While auxiliary treatments such as sonication or acidification are often used to overcome solubility barriers, these variations hinder the establishment of universal reaction protocols. Consequently, a critical research priority is to systematically standardize production methods to bridge the gap between biochemical processing conditions and the resulting biological activities of COS, thereby ensuring reproducible and clinically relevant therapeutic outcomes.

To overcome the inherent insolubility and limited biological utility of HMWC, we have systematically optimized hydrolysis protocols to precisely modulate its molecular weight. While conventional COS often exhibit suboptimal antimicrobial and antioxidant activities, recent evidence suggests that heterogeneous COS fractions around 1 kDa can elicit potent antibacterial and anti-inflammatory immune responses [19]. This functional disparity is likely attributable to specific oligomers, such as hexa-glucosamine [(GlcN)_6_: ~1 kDa], and to the technical challenges of precisely characterizing them. Under the hypothesis that AMC within the 1–3 kDa range possesses superior biological efficacy, we again emphasize the critical need for controlled enzymatic degradation of the purified HMWC. In this study, we refined an efficient production method to obtain structurally homogeneous AMC—a hydrolysate with a defined average MW of approximately 1.3 kDa—and rigorously validated its biological activity. Our findings offer significant academic and pharmaceutical insights, providing a robust framework for integrating AMC into diverse biomedical applications.

A critical limitation of traditional HMWC is its inherent insolubility at physiological pH, requiring acidic environments for dissolution that are often incompatible with clinical hydrogel formulations. By tailoring the MW and DD, we have engineered AMC_416_ to overcome these solubility constraints, ensuring its functionality and stability within pharmaceutical-grade hydrogels. While HMWC may exhibit certain biological activities in vitro, its high MW restricts its bioavailability and penetration. In contrast, AMC_416_ demonstrates superior relative bactericidal activity compared to its parent material. This enhanced efficacy, combined with its favorable solubility, provides a significant advantage in formulating potent therapeutic agents against resistant pathogens. This research contributes to the development of materials with potential therapeutic applications by exploring the relationship between the molecular structure of chitosan derivatives and their biological activity. By using AMC_416_ in hydrogel formulations, this study provides insights for the future development of materials with improved properties and potential uses in various fields.

## 2. Results and Discussion

### 2.1. Strategic Enzymatic Processing of HMWC to Generate Chitosan Hydrolysates with Potent Biological Activities

**Figure 1:**
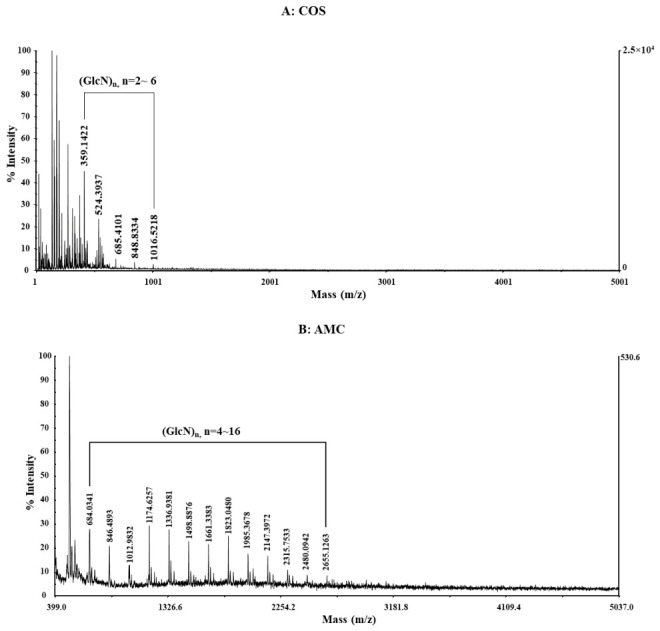
Structural characterization and mass analysis of enzymatically produced chitosan hydrolysates using MALDI-TOF spectrometry. The mass spectra are for the chitosan hydrolysate produced using free enzyme (**A**), and the mass spectra are for the chitosan hydrolysate produced by immobilized enzyme (**B**).

Establishing innovative methodologies to produce COS of defined MW from HMWC is a critical priority for advancing their industrial and pharmaceutical utilities. In this study, we addressed the limitations of conventional batch-type free enzyme systems [22,23]—which predominantly yield short-chain dimers and trimers (GlcN)_2~3_—by developing a column-type continuous hydrolysis system using immobilized enzymes. This shift in processing strategy is essential because higher degrees of polymerization, specifically single molecules larger than (GlcN)_6_ (approximately 1 kDa), have demonstrated superior biological activities, including potent anticancer effects, which are absent in lower-MW hydrolysates. As shown in Figure 1, MALDI-TOF mass spectrometry confirmed that while free enzymes maximize active site collisions to produce smaller fragments, the immobilized system limits substrate-enzyme contact, enabling the efficient production of targeted COS units ranging from (GlcN)_4_ to (GlcN)_16_. By bridging the gap between biochemical kinetic properties and standardized industrial production, this methodology provides a robust framework for manufacturing high-purity, bioactive COS for large-scale biomedical applications. Although we recognize the significance of the finding that COS molecules with a size of approximately 1 kDa (approximately 1007 Da when converted to (GlcN)_6_ exhibit potent anticancer effects [24], the efficient production of larger COS molecules is difficult to find, except for our previous study [19]. Therefore, we have strived to focus our research on the efficient production and characterization of single molecules larger than (GlcN)_6_.

In this regard, this study can be compared with the results of other researchers. There may be similarities in that (GlcN)_2_–(GlcN)_12_ COS-containing GlcN units were produced using hydrochloric acid, and their biological activity was evaluated [25]. However, this study highlights differences in terms of the design, efficiency, environmental friendliness, and mass production potential of the enzymatic treatment method for COS production, including antioxidant activity [26]. Furthermore, this is because structural differences in hydrolysates resulting from differences in the degree of deacetylation (DD) of HMWC can ultimately lead to changes in biological activity. To distinguish the unique molecular profiles of conventional COS and the active molecule chitosan (AMC), this study defined AMC as an oligomer fraction in the (GlcN)_4_–(GlcN)_16_ range. To differentiate AMC based on molecular weight distribution, the hydrolysate obtained in this study was named AMC_416_. While typical COS products have a molecular weight of less than 1 kDa, AMC_416_ maintains an average molecular weight of approximately 1.6 kDa, which is estimated to be a critical structural threshold directly associated with superior biological activity.

The industrial production and significance of AMC lie in establishing highly efficient, eco-friendly processes capable of mass-producing substances within specific molecular weight ranges. Moving forward, the evaluation of enzymatic activity must be approached from a new perspective: the productivity of bioactive molecules relative to pure enzymatic activity. We are currently refining the methods we have developed and utilized to evaluate diverse and unique biological activities. To further validate the clinical utility of AMC_416_, this study incorporated this active molecule into an AMC_416_-based hydrogel, confirming a significant enhancement in antimicrobial efficacy and an acceleration of anti-inflammatory activity in animal models. These results highlight the need for standardized and efficient AMC_416_ production as a foundational step toward developing high-performance biocompatible formulations for the future and beyond.

Although we used the same process as previous studies [19], the raw material, HMWC, cannot be standardized, so differences in MW and DD may occur depending on the conditions and environment of industrial production and batch to batch. However, since it is impossible to fully assess the effect of MW and DD on enzyme reactivity in the polymer state, we measured the average MW and/or DD of the products and used them as the basis for biological activity in subsequent studies.

### 2.2. Potent Antibacterial Efficacy of HMWC-Derived AMC_416_ Against Vancomycin-Resistant Staphylococcus aureus (VRSA)

The rising prevalence of VRSA underscores an urgent clinical demand for next-generation antimicrobial agents capable of circumventing established resistance mechanisms. While traditional methods—such as disk diffusion and liquid/solid culture assays—remain standard for evaluating antibacterial efficacy, their application to chitosan is frequently confounded by its inherent pH-dependency and high reactivity with extraneous culture media components. These biochemical limitations often obscure the accuracy of inhibitory data. To address these challenges, this study explores the correlation between the MW of chitosan and its targeted biological activity, specifically focusing on the inhibition of biofilm synthesis in VRSA. Furthermore, to bridge the gap between material synthesis and clinical utility, AMC_416_ was integrated into a hydrogel-based delivery system termed REOGEN (it is named to capture the industrial characteristics created with the keyword “organ regeneration”). This formulation leverages the structural advantages of hydrogels in pharmaceutical applications, providing a robust platform for the delivery of AMC_416_ as a potent therapeutic intervention against multidrug-resistant pathogens.

**Figure 2:**
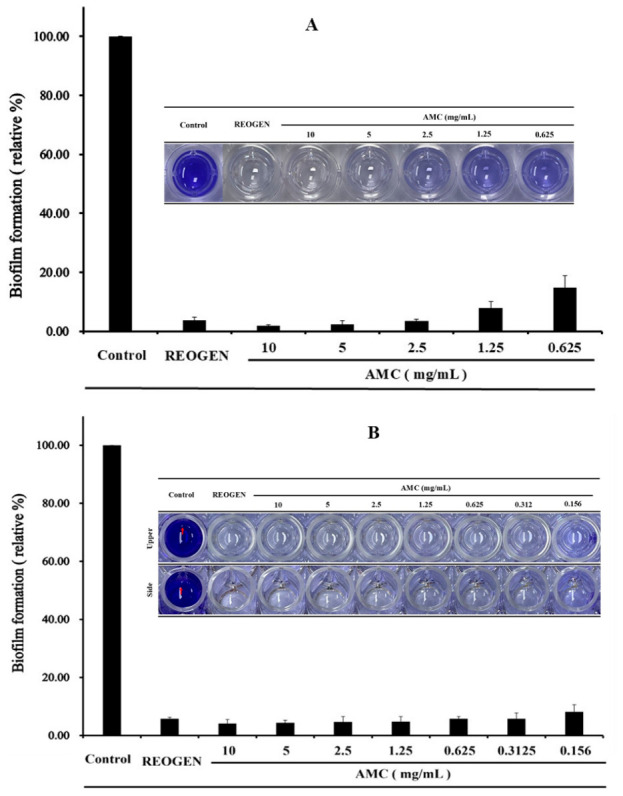
Characterization of the antimicrobial spectrum and potency of AMC_416_ against resistance-refractory VRSA. The antimicrobial activity of AMC_416_ against VRSA was assessed by examining its effects on biofilm formation inhibition (**A**) and the removal of adherent biofilms produced by VRSA growth (**B**). Acetic acid (1.0%, *v*/*v*) served as a control, while vancomycin (10 μg), a commercially available antibiotic, was used as a positive control. Additionally, an AMC_416_-based hydrogel called REOGEN was prepared. The concentration of AMC_416_ used was 5 mg/mL. Due to limited AMC_416_ availability, serial dilutions starting from 10 mg/mL AMC_416_ were employed to determine the effective concentration.

AMC_416,_ utilizing the molecular characteristics by the difference in MW of chitosan hydrolysates, also shows that anti-inflammatory activity and antibacterial efficacy using hydrogel formulations are suitable for inhibiting infection and promoting tissue regeneration in chronic wounds. Preliminary evaluations and established literature consistently demonstrate that AMC_416_ possesses significantly higher antibacterial activity than HMWC or COS at equivalent concentrations. In this study, HMWC—the parent material—was utilized as the primary control to eliminate potential analytical ambiguities associated with the molecular overlaps in COS. Experimental results confirmed that AMC_416_ inhibits VRSA biofilm synthesis in a robust, concentration-dependent manner (Figure 2A).

Beyond mere growth inhibition, AMC_416_ demonstrated a potent ability to reduce the adhesiveness of pre-established VRSA biofilms. As shown in Figure 2B, treatment with AMC_416_ led to significant biofilm detachment and structural degradation, underscoring its potential as a therapeutic agent for persistent, established infections. The AMC_416_-integrated hydrogel, REOGEN, exhibited highly significant antimicrobial activity. While previous studies have validated the general wound-healing properties of AMC_416_-based hydrogels, this study advances the clinical scope by evaluating their performance in artificially infected VRSA wound models. This transition from sterile wound healing to the management of multidrug-resistant infections represents a critical step in verifying the material’s immunological and histological efficacy in complex clinical environments. The findings emphasize that the biological activity of AMC_416_ is inextricably linked to its specific MW distribution. Consequently, for successful pharmaceutical translation, it is imperative to establish refined MW control technologies and optimized enzymatic reaction conditions to ensure the consistent and efficient production of a high-purity AMC molecule.

The therapeutic efficacy of chitosan is inherently dictated by its MW and the specific characteristics of the target pathogen. Recent research emphasizes that while lower MW ranges (11–30 kDa) are optimal for inhibiting Gram-negative bacteria like *E. coli*, Gram-positive strains such as *S. aureus* are more effectively suppressed as the MW increases within specific thresholds (e.g., below 300 kDa) [27,28]. Consequently, for successful pharmaceutical translation, it is imperative to move beyond traditional reactors in favor of advanced technologies—such as high-gravity systems and hydrodynamic cavitation—to achieve precise MW control, narrow polydispersity, and consistent bioactivity.

### 2.3. Concentration-Driven Growth Inhibition Kinetics of AMC_416_ Against Multidrug-Resistant VRSA

Current polymer-based hydrogels, such as those derived from cellulose and hyaluronic acid, are favored in clinical settings for their high biocompatibility and low toxicity. However, these materials primarily serve as passive scaffolds and lack inherent antimicrobial properties. This deficiency often leads to critical clinical failures, including an inability to prevent secondary infections and a lack of active signals required for rapid tissue regeneration. Consequently, their effectiveness in managing chronic or infected wounds remains insufficient, necessitating the development of next-generation biologically active dressings. Among natural biopolymers, chitosan stands out for its unique ability to disrupt microbial cell walls through cationic interaction while simultaneously promoting collagen synthesis and cell proliferation. Despite these advantages, its pharmaceutical translation is severely hindered by the challenge of selective MW control. The biological performance of chitosan is inextricably linked to its MW distribution [19,20]; however, traditional production methods often yield wide polydispersity and inconsistent purity, leading to unpredictable clinical outcomes.

**Figure 3:**
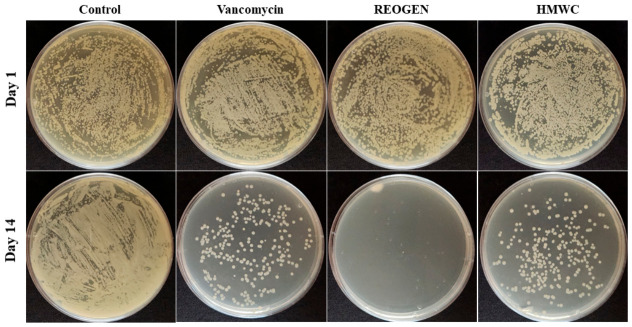
Evaluation of the antimicrobial potency of AMC_416_-incorporated hydrogel systems against pathogenic strains. Wounds were treated with a VRSA suspension at a concentration of 1 × 10^6^ cfu/mL. After air-drying, the wounds were treated with hydrogels containing vancomycin (10 µg/mL), REOEGN (5.0 mg/mL AMC_416_), and HMWC (5.0 mg/mL), respectively. After 1 and 14 days, the wounds were carefully cleaned with a sterile cotton swab and suspended in Luria–Bertani (LB) liquid medium. The suspensions were incubated at 37 °C for 24 h. 100 µL of each culture was plated onto LB solid medium and incubated at 37 °C for 24 h. The control group used a hydrogel of basic composition containing PBS.

To address the inherent inconsistency of natural polymers, we successfully optimized a selective MW control system utilizing an immobilized enzyme column under non-buffered conditions as described in the previous study [19]. This process ensures the efficient production of AMC within the precise 1–3 kDa range. This specific MW window is critical, as it maximizes the cationic interaction required for bactericidal activity while maintaining the molecular mobility necessary for penetration into complex biological matrices like biofilms. The justification for using AMC_416_ as a primary therapeutic agent is evidenced by its dual-action profile against VRSA. While the concentration required for 50% inhibition of biofilm formation (MIC_50_) was 1.25 mg/mL, the concentration required for biofilm removal was remarkably lower, at 0.312 mg/mL. This indicates that AMC_416_ does not merely prevent bacterial attachment but actively destabilizes established biofilm architectures, a functionality that traditional antibiotics and HMWC often lack. The most significant finding lies in the qualitative difference between transient growth inhibition and definitive bactericidal action. Comparative analysis revealed that both the vancomycin-treated and HMWC-treated groups allowed for bacterial survival and potential regrowth over time, as shown in Figure 3. The AMC_416_-integrated REOGEN hydrogel demonstrated sustained bactericidal properties for 14 days post-treatment, based on solid smear assays. This observed longevity of action in the experimental setting is a notable finding when considering potential clinical applications. The study suggests that the AMC_416_ within the hydrogel formulation may contribute to a prolonged antimicrobial effect in the tested model. This contrasts with the results seen in the vancomycin-treated group and the HMWC-treated group, where surviving VRSA were observed after the same period. Further investigation would be needed to understand the mechanisms behind this sustained activity and its clinical implications.

### 2.4. Promoting Cutaneous Wound Repair via the Application of REOGEN Hydrogel in Animal Models

**Figure 4:**
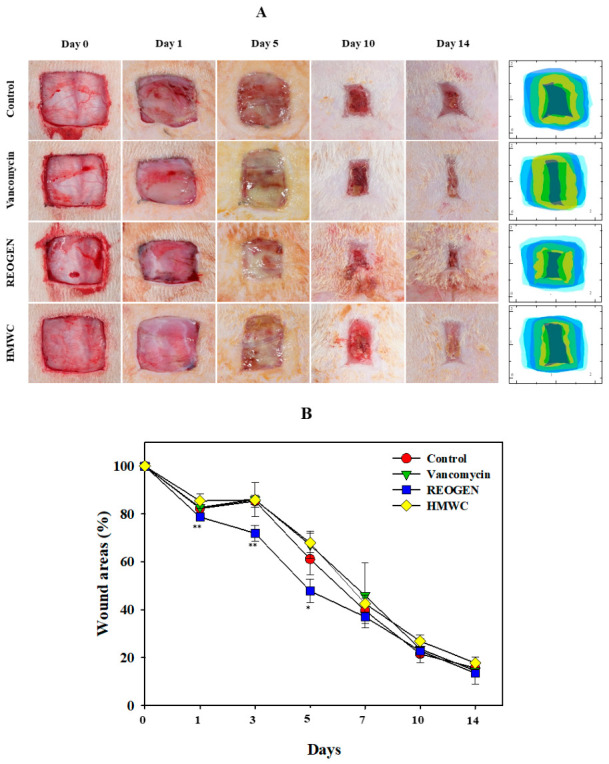
Evaluation of accelerated cutaneous regeneration in rats: highlighting differential healing kinetics across hydrogel treatment groups. In vivo treatment of a wound model mouse infected with VRSA (1 × 10^6^ CFU/mL) (**A**). Hydrogels containing PBS, vancomycin (10 μg/mL), REOGEN (containing 5 mg/mL AMC_416_), and HMWC (5 mg/mL) were applied to the wounds every 2 days, and wound images were taken for 14 days. Quantitative results of wound image area at various time points over time (mean ± SD, n = 3) (**B**). * *p* < 0.01, ** *p* < 0.001, compared with the control group. The control group used a hydrogel of basic composition containing PBS.

While prior studies suggested the wound-healing potential of heterogeneous AMC-based hydrogels in sterile or standard wound environments, they did not address the complexities of bacterial colonization. This study establishes a new benchmark by artificially inducing VRSA infections at the wound site, thereby simulating the challenging conditions of chronic, infected clinical wounds. The experimental results (Figure 4A,B) demonstrate that while HMWC provided only marginal healing, REOGEN—the AMC_416_-integrated hydrogel—yielded significant wound area reduction after 14 days. Notably, REOGEN’s performance was comparable to or exceeded that of vancomycin, despite the latter being a traditional “gold standard” for Gram-positive infections. This confirms that REOGEN does not merely act as a passive dressing but provides an active environment for tissue regeneration even in the presence of highly resistant pathogens. A key distinction of this research lies in its methodical approach to dosing. While vancomycin was applied at its established clinical concentration (10 μg/mL), the AMC_416_ concentration in REOGEN was strategically selected based on robust biofilm inhibition and bactericidal data derived earlier in the study. This provides a clear pharmacological rationale for using AMC_416_ as a viable alternative to conventional antibiotics, particularly in cases where antibiotic resistance renders standard treatments ineffective. The successful integration of potent antibacterial activity with rapid tissue regeneration in a VRSA-infected model underscores the clinical utility of REOGEN. Overall, the above study results suggest that AMC_416_-based hydrogels can be converted from experimental prototypes to pharmaceuticals for the management of chronic infectious wounds based on their anti-inflammatory activity and mitigation of secondary infections caused by VRSA.

### 2.5. Quantitative Immunohistochemical Analysis for Comparative Evaluation of Therapeutic Wound Healing Activity

**Figure 5:**
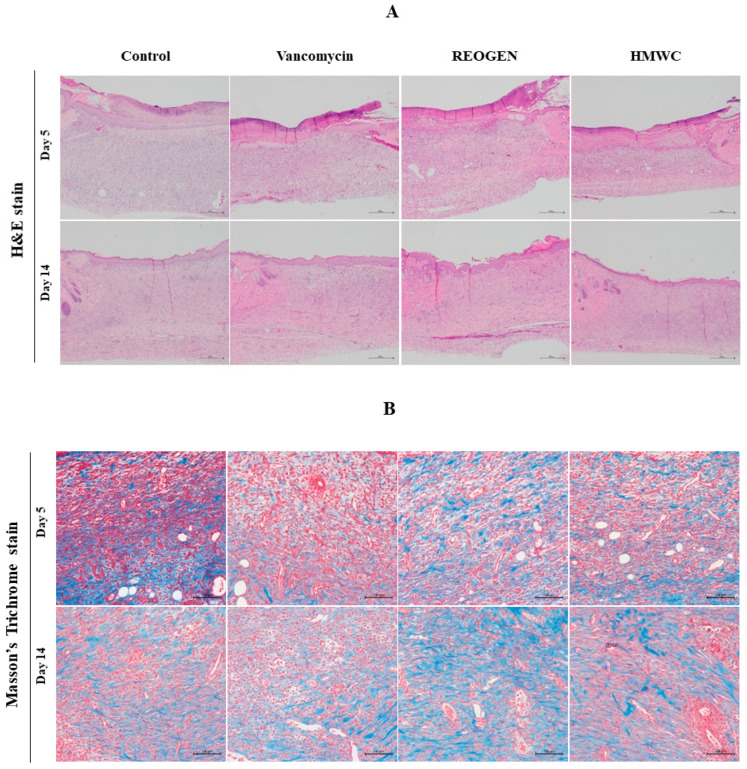

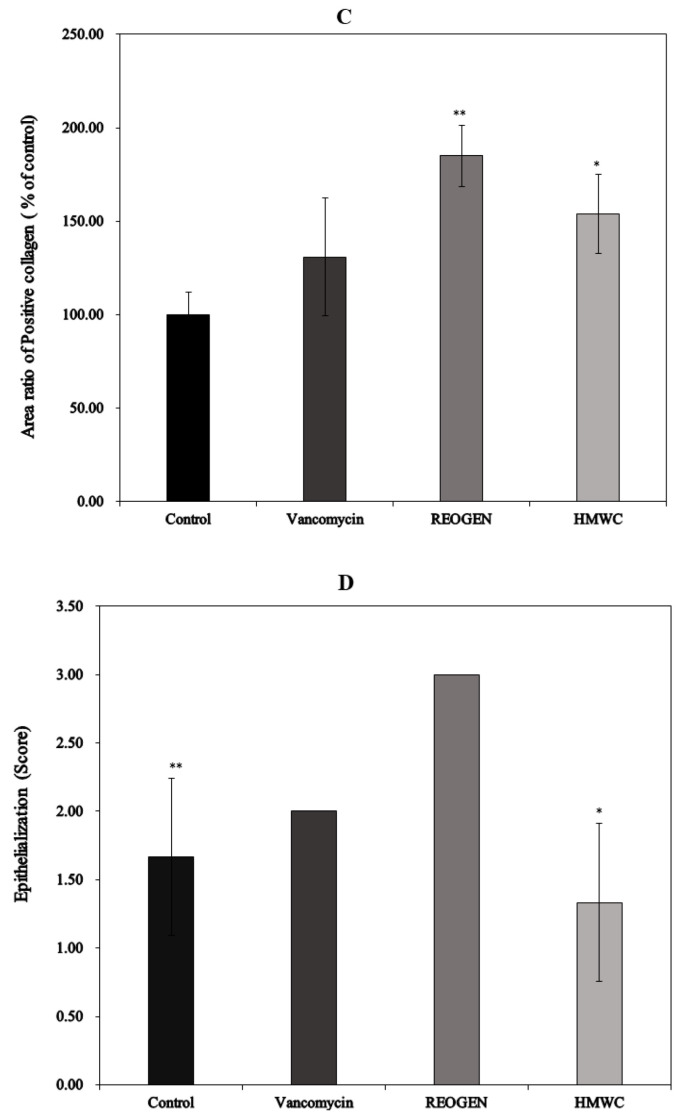
Comparative profiling of collagenous matrix formation in healed tissues via immunohistochemistry. Immunohistochemical staining was performed on tissue sections taken on days 5 and 14 after the infection with VRSA (1 × 10^6^ cfu/mL) and hydrogel constructs containing each composition. Hematoxylin & Eosin (H&E) staining of wound tissues showing overall tissue architecture and inflammatory cell infiltration (**A**). Masson’s trichrome staining revealed collagen deposition (blue staining) (**B**). Histopathological characteristics of skin wounds were observed at 100 (scale bar: 100 μm) magnification. Quantification of collagen deposition was performed by calculating the area ratio of positive collagen staining in Masson’s trichrome-stained sections (**C**). Epithelialization was assessed by measuring the percentage of wound area covered by new epithelium (**D**). Data are expressed as mean ± standard deviation (n = 5). * *p* < 0.05; ** *p* < 0.001.

Successful wound healing necessitates the precise coordination of keratinocyte and fibroblast activities to achieve re-epithelialization. However, in chronic lesions, this regenerative capacity is often severely impaired by persistent inflammation and diminished fibroblast function. Our immunohistochemical analysis (Figure 5) demonstrates that REOGEN significantly upregulates collagen synthesis, providing the essential structural framework required for cellular adhesion and proliferation. By reinforcing the extracellular matrix, the AMC_416_-based hydrogel effectively overcomes the inhibitory environment of chronic inflammation, facilitating the transition from the inflammatory phase to active tissue remodeling. The ability of REOGEN to simultaneously mitigate infection (as established in previous results) and promote high-density collagen deposition confirms its potential as a potent therapeutic intervention. This dual-action profile—combining potent antimicrobial activity with pro-regenerative signaling—addresses the primary failure points of conventional dressings in treating complex, non-healing wounds.

The hematoxylin–eosin (H&E) staining results provide critical evidence of the regenerative superiority of the REOGEN hydrogel (Figure 5A). In the presence of VRSA-induced inflammation, the REOGEN-treated group exhibited a notably thicker and more developed epithelial layer compared to both the vancomycin and HMWC groups. Furthermore, the presence of refined epithelial folding and increased tissue density serves as a histological hallmark of advanced re-epithelialization, suggesting that AMC_416_ actively promotes keratinocyte proliferation and migration even under pathological stress.

Masson’s trichrome staining further validates the functional quality of the regenerated tissue (Figure 5B). While all groups showed some degree of recovery, the REOGEN group demonstrated a significantly higher degree of uniform collagen deposition after 14 days. This homogeneous distribution of collagen fibers—characterized by intense blue staining—indicates a transition from disorganized granulation tissue to a structured extracellular matrix. Such architectural consistency is vital for restoring the mechanical strength of the skin and preventing the formation of hypertrophic scars. The significance of these histological observations lies in their confirmation that REOGEN does more than simply inhibit microbial growth. By fostering a thicker epithelial barrier and a more organized collagen network, REOGEN effectively reverses the cellular impairment typically caused by antibiotic-resistant infections. These findings position the AMC_416_-based hydrogel as a highly effective bioactive scaffold capable of orchestrating complex tissue remodeling, providing a definitive histological basis for its clinical application in treating infected chronic wounds.

### 2.6. Evaluating the Immunomodulatory Efficacy of AMC_416_ via Quantitative Analysis of Cytokine Dynamics in VRSA-Infected Models

**Figure 6:**
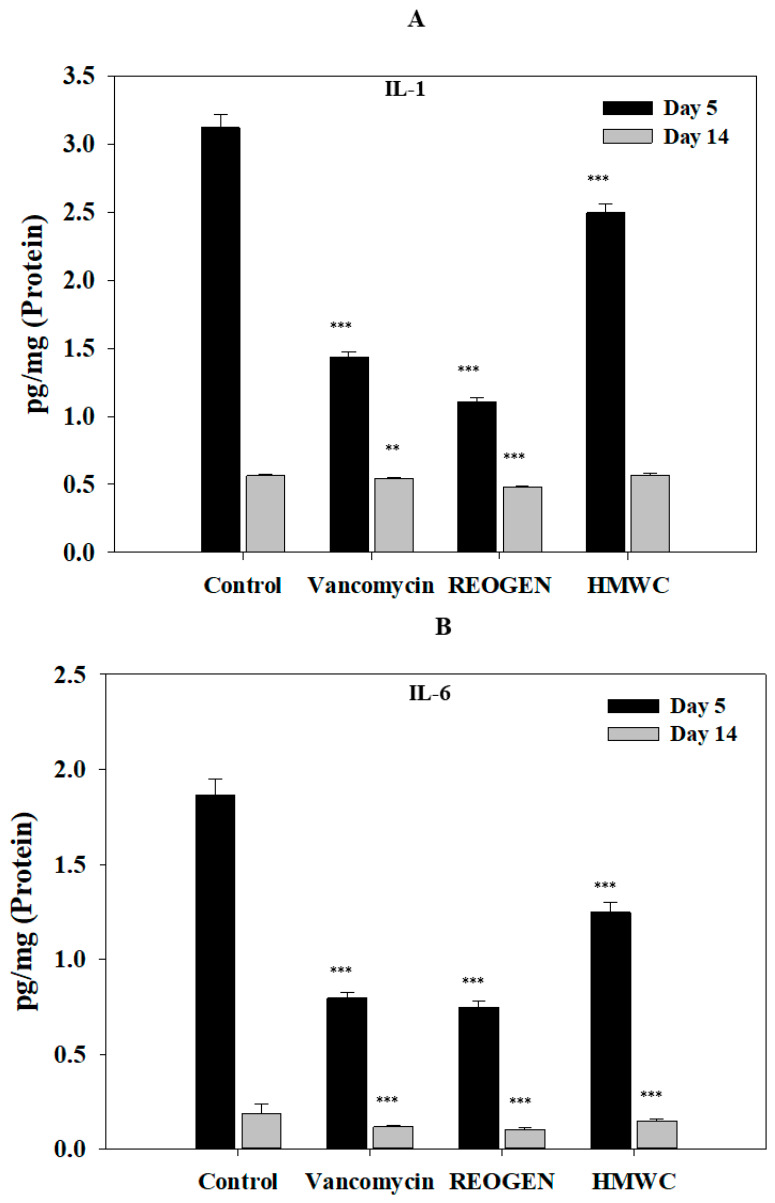

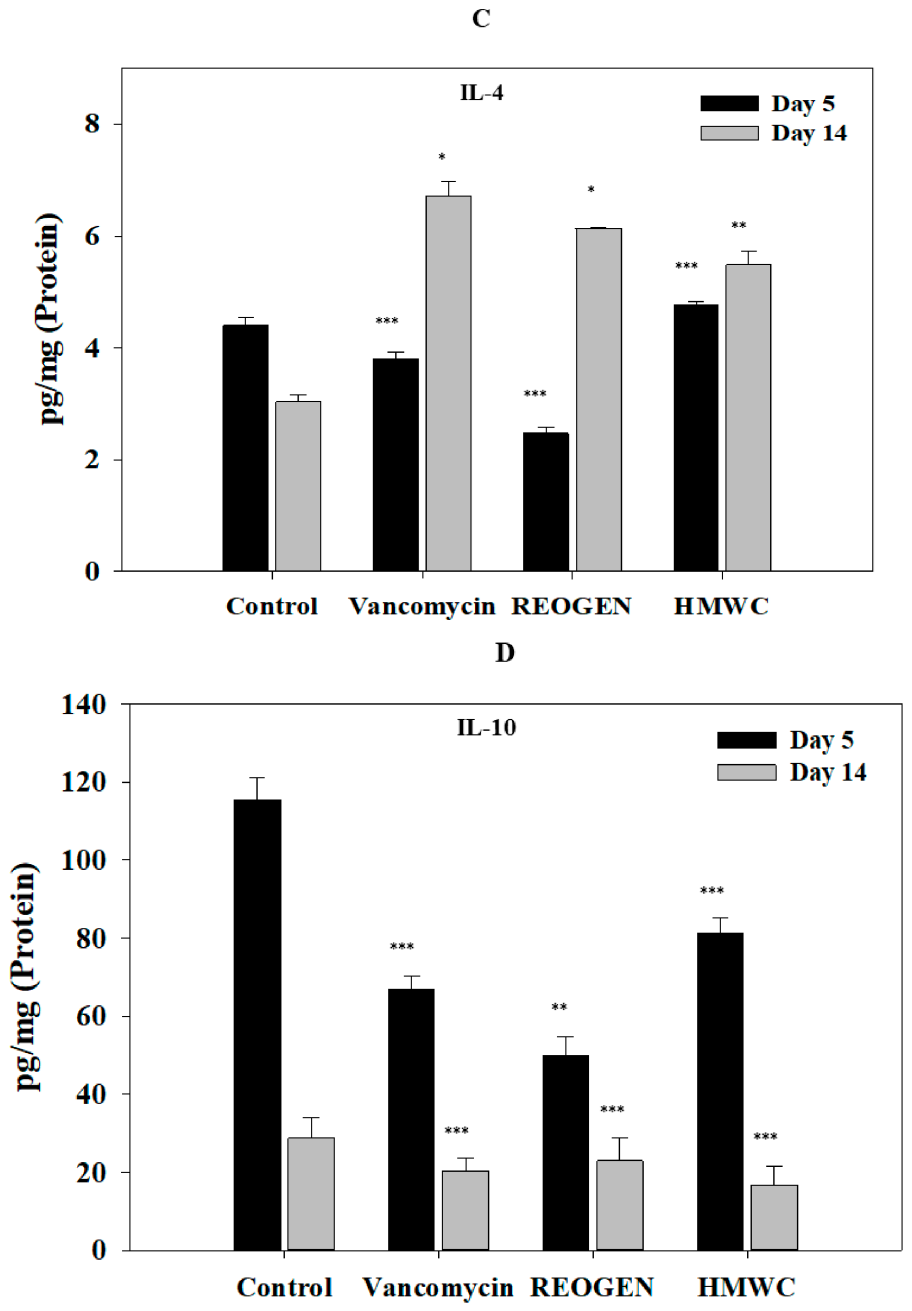
Modulation of the cytokine cascade in VRSA-infected tissues: a comparative study of pro- and anti-inflammatory mediators. Wounds were treated with 1 g of each hydrogel formulation at 2-day intervals after VRSA infection, and tissue samples were collected on days 5 and 14. Wound cytokine levels were measured using ELISA using protein extracted from the wound tissue. The measured cytokines were expressed in vivo as proinflammatory cytokines IL-1 and IL-6 (**A**,**B**), and anti-inflammatory cytokines IL-4 and IL-10. Data (mean ± standard deviation, n = 5) are expressed as pg cytokine per mg wound tissue (**C**,**D**). Statistical probabilities compared to the control group are indicated as * *p* < 0.05, ** *p* < 0.01, and *** *p* < 0.001, respectively.

The quantitative assessment of cytokines following VRSA infection (Figure 6A,B) reveals that REOGEN significantly attenuates the early-stage inflammatory surge. On day 5, the levels of IL-1 and IL-6 in the REOGEN-treated group were substantially lower than those in the control and other treatment groups. Given that IL-1 is a primary mediator of macrophage-driven immune activation and that IL-6 is a pivotal marker of the systemic inflammatory response, their rapid reduction suggests that AMC effectively mitigates the hyperinflammation typically triggered by multidrug-resistant pathogens such as VRSA. While IL-1 and IL-6 are essential for initial defense against injury and infection, their excessive or prolonged expression is a hallmark of chronic, non-healing wounds and autoimmune complications. The finding that REOGEN and Vancomycin-hydrogel groups achieved approximately a 50% reduction in IL-6 expression is of profound clinical importance. This modulation indicates that the AMC_416_-based hydrogel helps maintain the inflammatory response within a therapeutic window, preventing the tissue damage associated with cytokine overactivation while simultaneously neutralizing the bacterial threat. By day 14, cytokine levels across all groups stabilized toward baseline, but the early-phase intervention by REOGEN is the decisive factor in the overall healing trajectory. By resolving the acute inflammatory peak sooner than the control, REOGEN facilitates an accelerated transition into the proliferative phase of wound healing. This early immunological stabilization provides the necessary environment for the enhanced collagen synthesis and re-epithelialization observed in our histological data.

The significance of these results lies in the confirmation that REOGEN does not merely act as an antimicrobial agent but functions as an immunomodulator. Its ability to suppress excessive pro-inflammatory cytokine production during the critical early infection phase justifies its use as a sophisticated therapeutic for infected chronic wounds, where controlling the delicate balance of the immune response is as vital as eliminating the pathogen itself. A key finding in this study is the significant upregulation of IL-4 (Figure 6C), an anti-inflammatory cytokine critical for the transition to the remodeling phase of wound healing. While initial levels were consistent across groups on day 5, the REOGEN treatment group exhibited markedly higher IL-4 levels by day 14 compared to the vancomycin-treated group. This elevation suggests that AMC_416_ actively promotes Th2 cell differentiation and humoral immune responses, which are essential for modulating fibroblast activity and fostering a wound-healing microenvironment. The expression profile of IL-10 further highlights REOGEN’s efficiency in resolving acute inflammation (Figure 6D). In the control group, IL-10 levels spiked significantly during the early stage of infection—a reactive attempt by the body to suppress excessive inflammation. In contrast, the REOGEN group maintained lower, stable expression levels, indicating that the AMC_416_-based hydrogel had already neutralized the primary inflammatory stimuli. By day 14, the convergence of IL-10 to basal levels across groups confirmed the successful resolution of the inflammatory stage. This comprehensive immunological shift provides the biological justification for the superior epithelial tissue recovery and organized collagen synthesis observed in the H&E and MT histological analyses (Figure 5). These results confirm that REOGEN is not merely a passive barrier but a bioactive agent that orchestrates a favorable cytokine balance. By suppressing early-stage hyper-inflammation and sustaining late-stage regenerative signaling, the AMC_416_-based hydrogel establishes itself as a highly significant therapeutic for the treatment of complex, infected wounds where traditional antibiotic approaches often fail to address the underlying immune dysfunction. There is an increasing need to conduct various studies on specific aspects of secondary infection prevention and healing, such as dual-functional antimicrobial/angiogenic dressings [29] and regenerative medicine strategies in complex wound management [30]. Based on these results, the differences, uniqueness, and justification of the results of this study should be evaluated. This is because it is considered worthy of recognition as a follow-up study that complements the shortcomings of previous studies that were not addressed in detail.

To optimize the clinical and pharmaceutical trajectory of AMC_416_ in future studies, subsequent research must prioritize three pillars: elucidating the molecular signaling pathways (such as TGF-β/Smad or MAPK) driving fibroblast activation, conducting longitudinal safety and bio-distribution assays to ensure systemic compatibility, and initiating comparative human clinical trials for diverse etiologies like diabetic ulcers. By bridging the gap between sustained collagen synthesis and definitive clinical data, REOGEN establishes a robust framework for its adoption as a next-generation bioactive pharmaceutical for complex wound management.

## 3. Conclusions

This study presents a pioneering immobilized enzyme-packed column system capable of tight MW control (1–3 kDa), effectively addressing the inherent challenges of chitosan utilization due to its high MW. By efficiently producing chitosan hydrolysates with significant antimicrobial and anti-inflammatory activities, along with enhanced solubility and superior bioavailability, this platform offers a powerful biotechnological solution to the growing threat of multidrug-resistant pathogens worldwide. Furthermore, it establishes a rigorous biological framework for precision engineering of bioactive polymers, paving the way for standardized commercialization. Future research will focus on optimizing large-scale bioprocessing parameters to obtain high-purity, single-stranded AMCs, ultimately positioning the AMC_416_-based platform as a key technology for next-generation regenerative medicine and advanced antimicrobial therapeutics.

## 4. Materials and Methods

### 4.1. Materials

HMWC, a raw material for the production of COS or AMC, was purchased from Sokcho Trading Co. in Sokcho, Gangwon-do, Republic of Korea. HMWC with a DD of approximately 98% and an average MW of roughly 2000 kDa was used as the raw material for producing chitosan hydrolysates. Chitosanase derived from *Bacillus* species (EC 3.2.1.132) for COS or AMC production, along with glucose, GlcN, and GlcNAc for reducing sugar quantification to assess enzyme activity, was obtained from Sigma Chemical Co. (St. Louis, MO, USA). 2,5-dihydroxybenzoic acid (DHA), a matrix material for measuring the MW of COS or AMC via Matrix-Assisted Laser Desorption/Ionization Time-Of-Flight (MALDI-TOF) mass spectrometry, was purchased from Sigma Chemical Co. (St. Louis, MO, USA). The molecular porous membrane tube (MWCO 12–14 kDa) for material dialysis was acquired from Spectrum Lab, Inc., in Rancho Dominguez, CA, USA. Specific pathogen-free (SPF) rats, Hsd: Sprague Dawley^Ⓡ^™ SD^Ⓡ^™, were obtained from Coretech Co., Ltd. (406 Dongcheon-ri, Jinwi-myeon, Pyeongtaek-si, Gyeonggi-do, Republic of Korea). Tissue immunological examinations were performed at the Korea Nonclinical Technology Support Center in Seongnam, Gyeonggi-do, Republic of Korea. Tissue slides for immunohistological examination were imaged using an Eclipse TE2000-U microscope (Nikon, NI tech Bio Co., Ltd., Gyeonggi-do, Republic of Korea). Preclinical experiments involving wound healing and histological evaluation were conducted by HLB BioStep in Incheon, Republic of Korea, following the Animal Research Ethics Act. All other reagents used are ultra-pure substances.

### 4.2. Immobilized Enzyme Treatment for Chitosan MW Control

#### 4.2.1. Purification and Preparation of Chitosan Substrate

The basic protocol was slightly modified and performed as follows. Briefly, 15 g of HMWC (DD 98%, <100 mesh) was added to a 50% NaOH solution (100 mL) and deacetylated by heating in a double boiler using cooking oil maintained at 120 °C for 12 h. After completion of the reaction, chitosan was filtered through a filter paper when the temperature reached approximately 30–40 °C. Subsequently, it was washed with a large amount of water to remove impurities and residual NaOH. After washing, it was freeze-dried at −50 °C (TFD8503, Ilshin, Republic of Korea). The preparation and purification of the chitosan solution for enzyme treatment were performed according to this method [31]. For the enzymatic substrate preparation, the purified HMWC was dissolved in 2% (*w*/*v*) acetic acid and subsequently diluted with 50 mM sodium acetate buffer (pH 5.0) at a volumetric ratio of 1:4 (*v*/*v*) to achieve optimal reaction conditions.

#### 4.2.2. Production of COS via Batch Reaction

COS was synthesized through a batch enzymatic hydrolysis process. Commercial chitosanase (2.0 U) was introduced as a free enzyme into 10 mL of the prepared chitosan solution. The mixture was incubated at 37 °C with constant agitation. To monitor the hydrolysis kinetics, aliquots were collected at 2 h intervals. The reaction endpoint was determined by quantifying the concentration of reducing sugars, identifying the time point at which no further increase in enzymatic liberation of reducing ends was observed [20].

#### 4.2.3. Synthesis of AMC via a Continuous Immobilized Enzyme System

AMC was produced using a continuous flow reactor system utilizing immobilized chitosanase, as described by Lee et al. [19]. It consisted of 3 steps: (1) *Desalination and buffer exchange*: 50 mL of the 2% (*w*/*v*) chitosan solution was subjected to primary dialysis using a membrane with a molecular weight cut-off (MWCO) of 12–14 kDa to remove residual solvents. A secondary dialysis was performed against 1 L of 50 mM sodium acetate buffer (pH 5.0) for equilibration. (2) *Column chromatography and reaction*: The immobilized chitosanase (2 U) was packed into a cylindrical glass column (1.0 cm i.d. × 8 cm). The chitosan substrate was delivered through the immobilized enzyme bed at a constant flow rate of 1 mL/min using a calibrated peristaltic pump (Innofluid Co., Ltd. Shanghai, China). (3) *Sampling and quantitative analysis*: Effluent samples (100 µL) were collected every 2 h over 24 h and stored at −20 °C for subsequent analysis. The degree of HMWC degradation and the reaction efficiency were evaluated by the 4-hydroxybenzhydrazide (4-PHBAH) method [20]. Absorbance was measured at 405 nm using an Infinite M200 Pro Nano-Quant microplate reader (Tecan Austria GmbH, Grödig, Austria). D-glucosamine (GlcN) served as the external standard for the quantification of reducing sugar equivalents.

### 4.3. Application of MALDI-TOF Mass Spectrometry for the Identification of COS and AMC

The MW of both COS and AMC was determined via a matrix-assisted laser desorption/ionization time-of-flight (MALDI-TOF-MS) mass spectrometer (Bruker Autoflex Speed, Billerica, MA, USA) following the method of Lee et al. [13]. Mass spectra were acquired in positive reflector mode (*m*/*z* 400–5000) using a 355 nm Nd:YAG laser (200 Hz) and an accumulation of 1050 shots per spectrum. The system was calibrated with a TOF/TOF mixture, and data were analyzed using Data Explorer software (Voyager, AB SCIEX, Framingham, MA, USA). Product MWs were calculated based on the *m*/*z* values of GlcN and GlcNAc units. The molecular weights of these chitosan hydrolysates were measured by MALDI-TOF mass spectrometry. The mass range was set from 599 to 2500 *m*/*z*, and the relative masses were measured using 2,5-dihydroxybenzoic acid (DHA) as the base matrix. These results were provided by the Seoul National University Joint Research Institute.

### 4.4. Inhibition and Removal of Biofilm Formation by AMC_416_

#### 4.4.1. Biofilm Inhibition Assay (Formation Phase)

To assess the prophylactic potential of AMC416, Vancomycin-Resistant *Staphylococcus aureus* (VRSA) (1 × 10^6^ CFU/mL) was inoculated into 96-well plates to induce biofilm formation. The base hydrogel (AMC-free) used as a control contained the following: AMC 5%, (Excipient) Glycerin 8% (DUKSAN Chemicals, Ansan-si, Republic of Korea), (Thickening Agent) Hydroxyethyl-cellulose 1.0% (Ashland, Wilmington, DE, USA), (Solubilizer) PEG-60 Hydrogenated Castor Oil 0.02% (Covix, Inchon, Republic of Korea), (Solvent) Distilled Water 85.98%. The inhibitory capacity was evaluated by treating the inoculum with a concentration gradient of AMC416 (0.625 to 10 mg/mL). Simultaneously, the functional stability of AMC416 (5 mg/mL) was tested by incorporating it into the REOGEN hydrogel (in this study, a specific composite hydrogel formulated with AMC416 as the primary active pharmaceutical ingredient is designated as ‘REOGEN’). After 48 h of incubation at 37 °C, biomass was quantified via crystal violet staining. Following three washes with distilled water, the stained biofilm was solubilized in 0.2 mL of ethanol, and optical density (OD) was measured at 680 nm to calculate relative inhibition compared to untreated controls.

#### 4.4.2. Biofilm Eradication Assay (Mature Phase)

To determine the efficacy against pre-established biofilms, VRSA (1 × 10^8^ CFU/mL) was first incubated for 48 h to allow for complete matrix maturation. After the removal of the supernatant, the mature biofilm was exposed to varying concentrations of AMC_416_ (0.156 to 10 mg/mL) and AMC_416_-loaded REOGEN (5 mg/mL) for 8 h at 37 °C. Residual biomass was analyzed using the 4-hydroxybenzhydrazide-linked crystal violet method. Absorbance was recorded at 680 nm, and the results were expressed as a percentage of biofilm removal relative to the control group (medium-only treatment) to validate the therapeutic potential of the AMC_416_-hydrogel system.

### 4.5. Investigation of the Wound-Healing Efficacy of AMC_416_-Functionalized Hydrogels Through Targeted Anti-Inflammatory Action

This study validates the antibacterial and therapeutic potential of REOGEN, an AMC_416_-integrated hydrogel, against VRSA. Beyond assessing the intrinsic activity of AMC_416_, the research focuses on its functional stability within a multicomponent pharmaceutical matrix, confirming sustained bactericidal efficacy by suppressing colony formation over 14 days. The hydrogel formulations, optimized for biocompatibility, gelation kinetics, and thermal stability, were compared with Vancomycin and HMWC controls. Quantitative analysis of wound closure (mean ± SD, n = 3) further demonstrated that REOGEN maintains potent antimicrobial activity while significantly accelerating healing, establishing its efficacy as a safe, stable topical agent for resistant infections.

### 4.6. Validation of Wound Healing Promotion by AMC_416_-Incorporated Hydrogels: An Animal Study Focused on Anti-Inflammatory Mechanisms

To evaluate the therapeutic potential of the test substances, this study utilized a robust Sprague–Dawley (SPF) rat model to simulate both surgical and VRSA-induced infectious wounds, a methodology that holds significant academic weight by addressing the critical challenge of multidrug-resistant pathogen management in regenerative medicine. SPF rats, Hsd: Sprague Dawley^Ⓡ^™ SD^Ⓡ^™, were purchased from Coretech Co., Ltd. (406 Dongcheon-ri, Jinwi-myeon, Pyeongtaek-si, Gyeonggi-do, Republic of Korea). Acclimation of the mice was performed in a controlled environment at 22 ± 3 °C with a relative humidity of 50 ± 10% under a 12 h light/dark cycle for one week. “All animal procedures were approved by the Institutional Animal Care and Use Committee (IACUC) of HLB BioStep Co., Ltd. (Approval No. 24-HB-0343) and were performed in accordance with relevant guidelines and regulations.” A key procedural specificity lies in the dynamic administration of the viscous test substance (1 mL), which was strategically applied nine times over 14 days to ensure sustained pharmacological contact and functional stability within the infected microenvironment. Furthermore, the use of longitudinal ImageJ (v1.53)-based morphometric analysis—while carefully preserving the physiological integrity of the wound by leaving scabs intact when necessary—allowed precise, non-invasive quantification of the wound-closure trajectory. This integrated approach not only validates the biocompatibility and safety of the formulation but also provides essential preclinical evidence of its ability to overcome the inhibitory effects of bacterial biofilms on the natural healing process.

### 4.7. Characterization of Tissue Architecture and Inflammatory Response During Hydrogel-Induced Wound Healing

To evaluate the dynamic wound-healing process, necropsies were performed in two stages: an interim assessment on Day 5 (n = 2 per group) and a final evaluation on Day 14 (remaining animals). The excised wound sites, including adjacent normal tissues, were bifurcated for multifaceted analysis, with one half cryopreserved at −80 °C and the other fixed in 10% neutral buffered formalin for histopathological examination. Formalin-fixed specimens underwent standardized processing—including trimming, dehydration, and paraffin embedding—and were subsequently analyzed using Hematoxylin & Eosin (H&E) for structural morphology, Masson’s Trichrome (MT) for collagen deposition, and Immunohistochemistry (IHC) for inflammatory markers (IL-10 and TNF-α). Quantitative scoring was conducted under an Olympus BX53 light microscope according to the validated criteria of Nisbet et al. [31], while MT-stained images were digitized via a Carl Zeiss Axio Scan.Z1 and quantified using ZEN software (ZEN 2.3) to ensure a statistically robust and integrated assessment of therapeutic efficacy (Carl Zeiss ZEN, Oberkochen, Germany).

### 4.8. Immunomodulatory Analysis of Anti-Inflammatory Cytokine Expression in Response to VRSA-Triggered Inflammation

The quantitative evaluation of pro-inflammatory (IL-1ß, IL-6) and anti-inflammatory (IL-4, IL-10) cytokines is a critical prerequisite for validating the immunomodulatory efficacy of the developed hydrogels in VRSA-infected wounds. Given that multidrug-resistant infections often trigger a dysregulated and prolonged inflammatory phase that stalls the regenerative process, monitoring these specific biomarkers is essential to demonstrate the transition from a pathological inflammatory state to an active healing trajectory [19]. To achieve this, 1 g of each hydrogel formulation was administered biannually following wound induction, with target tissues harvested at strategic intervals (Days 5 and 14) to capture the kinetic shifts in the cytokine profile. Proteins were precisely extracted from the excised tissues and quantified via high-sensitivity Enzyme-Linked Immunosorbent Assays (ELISA) in strict accordance with standardized protocols. By meticulously balancing the ratio between pro- and anti-inflammatory mediators, this analysis provides pivotal evidence of the hydrogel’s ability to resolve chronic inflammation and re-establish a pro-regenerative microenvironment in the presence of highly resistant pathogens.

### 4.9. Statistical Analysis

To ensure the reproducibility and statistical robustness of the findings, all experimental procedures were performed in at least triplicate. Quantitative data are presented as the mean ± standard deviation (S.D.), reflecting the precision of the observed outcomes. Statistical significance was rigorously evaluated to determine the efficacy of the treatments compared to the control group. Differences were considered statistically significant at *p*-values of less than 0.05 (* *p* < 0.05), 0.01 (** *p* < 0.01), or 0.001 (*** *p* < 0.001), while values of *p* ≥ 0.05 were regarded as not significant. This hierarchical approach to *p*-value designation allows for the precise interpretation of the biological impact and the statistical confidence of the AMC-integrated hydrogel’s therapeutic performance.

## Data Availability

The data presented in this study are available on request from the corresponding author due to proprietary reasons or pending patent applications.
